# Evaluation of Multiple Semi-Twisted Tape Inserts in a Heat Exchanger Pipe Using Al_2_O_3_ Nanofluid

**DOI:** 10.3390/nano11061570

**Published:** 2021-06-15

**Authors:** Yongfeng Ju, Tiezhu Zhu, Ramin Mashayekhi, Hayder I. Mohammed, Afrasyab Khan, Pouyan Talebizadehsardari, Wahiba Yaïci

**Affiliations:** 1 Faculty of Electronic Information Engineering, Huaiyin Institute of Technology, Huai’an 223003, China; jinmulu@163.com; 2 Young Researchers and Elite Club, Khomeinishahr Branch, Islamic Azad University, Khomeinishahr 119/84175, Iran; ramin.mashayekhi.me@gmail.com; 3 Department of Physics, College of Education, University of Garmian, Kurdistan, Kalar 46021, Iraq; hayder.i.mohammad@garmian.edu.krd; 4 Institute of Engineering and Technology, Department of Hydraulics and Hydraulic and Pneumatic Systems, South Ural State University, Lenin Prospect 76, 454080 Chelyabinsk, Russia; khana@susu.ru; 5 Centre for Sustainable Energy Use in Food Chains, Institute of Energy Futures, Brunel University London, Kingston Lane, Uxbridge, Middlesex UB8 3PH, UK; 6 CanmetENERGY Research Centre, Natural Resources Canada, 1 Haanel Drive, Ottawa, ON K1A 1M1, Canada

**Keywords:** twisted tape inserts, nanofluid, performance evaluation criterion, heat exchanger pipe, Nusselt number, friction factor

## Abstract

The hydrothermal performance of multiple semi-twisted tape inserts inside a heat exchanger pipe is numerically examined in three-dimensions. This study aims to find the optimum case for having the highest heat transfer enhancement with the lowest friction factor using nanofluid (Al_2_O_3_/water). A performance evaluation criterion (PEC) is defined to characterize the performance based on both friction factor and heat transfer. It was found that increasing the number of semi-twisted tapes increases the number of swirl flow streams and leads to an enhancement in the local Nusselt number as well as the friction factor. The average Nusselt number increases from 15.13 to 28.42 and the average friction factor enhances from 0.022 to 0.052 by increasing the number of the semi-twisted tapes from 0 to 4 for the Reynolds number of 1000 for the base fluid. By using four semi-twisted tapes, the average Nusselt number increases from 12.5 to 28.5, while the friction factor reduces from 0.155 to 0.052 when the Reynolds number increases from 250 to 1000 for the base fluid. For the Reynolds number of 1000, the increase in nanofluid concentration from 0 to 3% improves the average Nusselt number and friction factor by 6.41% and 2.29%, respectively. The highest PEC is equal to 1.66 and belongs to the Reynolds number of 750 using four semi-twisted tape inserts with 3% nanoparticles. This work offers instructions to model an advanced design of twisted tape integrated with tubes using multiple semi-twisted tapes, which helps to provide a higher amount of energy demand for solar applications.

## 1. Introduction

The increasing energy demand will require improving the energy efficiency of heat transfer applications [1,2]. Due to high energy demand, renewable energies, especially solar energy, have been widely employed in recent decades [3,4]. Collectors are one of the solar energy technologies that have been widely employed to convert solar energy into useful thermal energy [5,6,7]. In the collector, the solar radiation is reelected to the absorber tube and provides an almost uniform heat flux around the tube’s wall [8,9,10]. There are several techniques in the literature working on heat transfer enhancement into the heat transfer fluid (HTF) inside the tube, including the use of inserts and modifying the characteristics of the HTF [11,12,13]. 

Inserts have been attracted lots of attention, though not regarding their effect on the characteristics of the employing fluid [8,14]. There are different types of inserts inside the tubes to increase the heat transfer to the working fluid, including fins, twisted tapes, porous discs, turbulators, perforated plates, and dimples, etc. [15,16,17,18,19]. Twisted tapes have been widely employed inside the tubes to increase the heat transfer performance in the tube and have shown less effect on the pressure drop compared with other enhancement techniques such as fins [20,21,22,23]. In a tube integrated with twisted tape inserts, swirl flow is generated with higher axial fluid velocity along the tube, resulting in a higher heat transfer [24,25,26,27]. Furthermore, twisted tapes provide a mixing flow similar to a turbulator, which helps heat transfer enhancement [27,28,29,30]. There are different studies in the literature to increase the performance of twisted tapes inside the tubes that have been performed both numerically and experimentally [31,32,33,34].

Lim et al. [35] investigated experimentally a twisted tape inserted tube in a laminar flow regime using variable and constant pumping power. They showed a high contribution of twisted tape inserts to the performance of the heat exchanger. The results showed that the friction factor and Nusselt number were enhanced by factors of 10 and 3, respectively. Jaramillo, et al. [20] assessed a parabolic through a collector equipped with twisted tape. They claimed that the thermal performance of the collector increases as the twisted ratio reduces at low Reynolds numbers. They proved the higher performance of the PTC collector using a twisted tape insert in a passive way. Mwesigye et al. [36] studied a parabolic through a collector with wall-detached twisted tape. They indicated that higher values of twist ratio and lower values of width result in the enhancement of the optimal Reynolds number. Sajadi et al. [37] performed an experimental study on the heat transfer and pressure drop of R123yf condensation flow inside a tube with twisted tape inserts compared with a plain tube. They showed that, in a twisted tape inserted tube compared with a plain tube, 42% and 235% enhancement occurred in the heat transfer coefficient and the pressure drop, respectively. They claimed the maximum heat transfer coefficient occurs in a twist ratio of 6, and the maximum overall enhancement ratio happens in a twist ratio of 9. Kurnia et al. [38] investigated numerically the twisted tape inserts in a helical tube. They showed that the heat transfer enhances by up to four times in a twisted tape inserted helical tube compared with the straight tube with a twisted tape insert. However, the pressure drops also increase in a helical tube. Aliabadi et al. [39] studied the twisted tape inserts in a twisted tube compared with a straight tube and showed that the combination of twisted tape and twisted tubes has a significant influence on improving the performance of the model. They showed the maximum performance efficiency coefficient of 3.21 for the best scenario. Furthermore, a higher performance is achieved with a higher twisted pitch.

There are limited studies in the literature incorporating a higher number of twisted tape inserts in a tube in the forms of separated and joint twisted tapes [40]. Dalkilic et al. [41] examined the effect of quad twisted tape inserts in a tube experimentally using hybrid nanofluid for different lengths. They showed that a higher Nusselt number and friction factor could be achieved by increasing the length of the tape inside the tube. He et al. [42] examined the effects of using double separate twisted tapes in a tube compared with a single tape under turbulent fluid flow conditions using nanofluid. They showed that the maximum performance efficiency coefficient for the case of single twisted tape is almost 6.5% higher than that for a double twisted tape, which is defined based on the Nusselt number over the friction factor. Bhuiya et al. [43] studied the hydrothermal characteristics of a perforated twisted tape inserted tube experimentally using triple joint twisted tapes. They showed that the rate of heat transfer enhances by 88–320%, while the friction factor enhances by 112–355% for different porosities of the perforated tubes. The highest Nusselt number and friction factor was achieved in the porosity of 4.6%.

Nanofluid has been recently widely employed in various heat transfer applications to improve the thermophysical properties of the working fluid toward higher heat transfer performance [19,22,44,45,46,47]. Regarding twisted tape inserted tubes, there are several studies on the simultaneous usage of nanofluid and twisted tapes for a higher rate of heat transfer [12,48,49,50,51]. Qi et al. [52] investigated the convective nanofluid heat transfer in a tube using rotating and static built-in twisted tape elements, experimentally. They reported 101.6% enhancement in heat transfer by using rotating twisted tape inserts along with the nanofluid. In another empirical study, Sunder et al. [53] examined the thermal performance of a solar water heater employing nanofluid and twisted tape inserts as heat transfer enhancement techniques. They showed 49.75% improvement using the best configuration of twisted-tape. Sheikholeslami et al. [54] examined the effect of CuO/water nanofluid in a twisted tape inserted tube in a turbulent flow regime based on the first and second laws of thermodynamics [55]. They showed a higher Bejan number for a higher twist pitch ratio. Furthermore, higher entropy generation was gained for a lower Nusselt number.

According to the presented literature review, there are limited studies on the use of multiple twisted tape inserts in a tube, especially in the presence of nanofluid. Furthermore, there is a limited comparative investigation on the performance of multiple semi-twisted tape inserts in a tube [56,57,58]. Therefore, in this study, the hydrothermal performance of multiple semi-twisted tapes integrated inside a tube is examined to find the optimum case for having the highest heat transfer enhancement with the lowest friction factor. An Al_2_O_3_/water nanofluid is used to improve the properties of the working fluid toward a higher heat transfer efficiency using a two-phase mixture model. Both the Nusselt number and friction factor are studied to provide a comparative study on the advantages of heat transfer enhancement and the disadvantages of pressure drop penalty. The effect of the Reynolds number is also investigated. This study points out some hydrothermal aspects, such as the tangential and radial velocities of the nanofluid, which has been rarely discussed in the literature. It should also be mentioned that, in this study, the use of one semi-twisted tape is introduced for the first time. This study provides guidelines to design an improved configuration of twisted tape inserted tubes using multiple semi-twisted tapes. The more efficient usage of twisted tape inserted tubes could help to provide a higher amount of energy transmission suitable for application in parabolic trough solar collectors.

## 2. Problem Statement 

**Figure 1:**
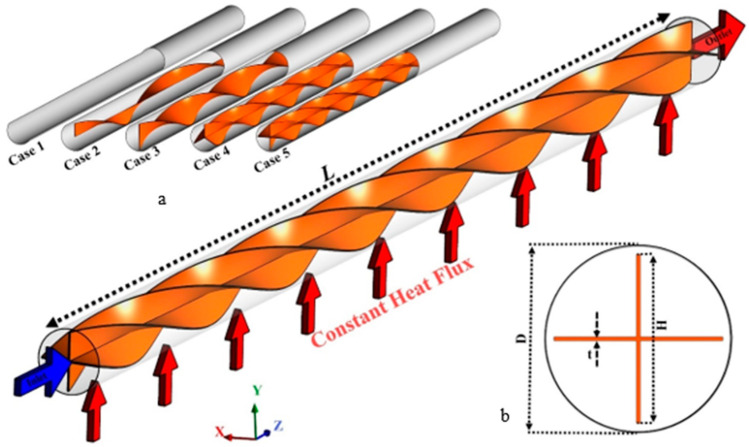
Schematic of the studied system using multiple semi-twisted tape inserts. (**a**) different proposed cases, (**b**) Boundary conditions and dimensions of the studied system.

The schematic of the proposed system under investigation is presented in Figure 1. The combination of a tube integrated with multiple semi-twisted tapes is investigated using different numbers of one, two, three, and four tape strips named as Cases 2 to 5 (Figure 1a). Case 1 is the tube without any twisted tape inserts. The diameter (D) and length (L) of the tube, as well as the thickness (ε) and height (H) of the twisted tape are 20 mm, 400 mm, 0.4 mm, and 19 mm, respectively, shown in Figure 1b. The flow enters the tube at the temperature of 300 K with different Reynolds numbers, while constant heat flux is applied around the tube.

Figure 1 schematically shows the counter flow double-pipe system equipped with overlapped twisted tapes in inner and outer tubes. The inner and outer tube diameters are 10 and 29 mm, respectively, and the thickness of the twisted tape is 0.4 mm with equivalent pitches of 100 mm. Two models for embedding the overlapped twisted tapes are considered; in the first model, the inner and outer twisted tapes swirl in the same angular direction (Co-STT as an abbreviation of co-swirling twisted tapes) and in the second model, the inner and outer twisted tapes swirl in the opposite angular direction (Counter-STT as an abbreviation of counter-swirling twisted tapes). The plain heat exchanger (PHE) is also studied, compared with the twisted tape cases. Al_2_O_3_-water nanofluid enters the inner and outer tubes at a temperature of 300 K, considering four different Reynolds numbers. Alumina (Al_2_O_3_) is among the more common and cheaper nanoparticles utilized in different commercial and experimental applications and has been well reported by a number of researchers. The alumina/water nanofluid shows strong enhancement on the thermophysical properties of the base fluid [59]. Moreover, Al_2_O_3_ nanoparticles have other advantages, such as chemical and thermal stability and excellent dispersion properties in the base fluid [60].

## 3. Mathematical Model

The present work will investigate a 3D steady-state laminar flow of incompressible Al_2_O_3_-water nanofluid, neglecting the effects of radiation and viscosity losses. The two-phase mixture model [61] is employed to model Al_2_O_3_ nanoparticles dispersed in water. The governing equations are defined as follows [62]:

- Continuity equation:

(1) $$\nabla .\left(\rho_{m}{\vec{V}}_{m}\right)=0$$

- Momentum equation:

(2) $$\vec{\nabla }.\left(\rho_{m}{\vec{V}}_{m}{\vec{V}}_{m}\right)=-\vec{\nabla }p+\vec{\nabla }\cdot \left[\mu_{m}\left(\vec{\nabla }{\vec{V}}_{m}+\vec{\nabla }{\vec{V}}_{m}^{T}\right)\right]+\rho_{m}\vec{g}+\vec{F}-\vec{V}\cdot \left(\sum_{k=1}^{n}\phi_{k}\rho_{k}{\vec{V}}_{\mathrm{dr},k}{\vec{V}}_{\mathrm{dr},k}\right)$$

where the secondary phase drift velocity is obtained from [27]:

(3) $${\vec{V}}_{\mathrm{dr},k}={\vec{V}}_{k}-{\vec{V}}_{m}$$

- Energy equation:

(4) $$\vec{\nabla }\cdot \left[\overset{n}{\underset{k=1}{\sum }}\left(\rho_{k}c_{pk}\right)\phi_{k}\vec{V_{k}}T\right]=\vec{\nabla }\cdot k_{m}\vec{\nabla }T$$

where *V_m_* and *ρ_m_* are given as:

(5) $${\vec{V}}_{m}=\frac{\sum_{k=1}^{n}\phi_{k}\rho_{k}{\vec{V}}_{k}}{\rho_{m}}$$

(6) $$\rho_{m}=\sum_{k=1}^{n}\phi_{k}\rho_{k}$$

In Equation (5), the subscription k is related to the kth phase of the mixture. ${\vec{V}}_{pf}$ and $\mu_{m}$ are given as [34]:

(7) $${\vec{V}}_{pf}={\vec{V}}_{p}-{\vec{V}}_{f}$$

(8) $$\mu_{m}=\overset{n}{\underset{k=1}{\sum }}\phi_{k}\mu_{k}$$

The equation developed by Manninen [63] and Shiller–Newman’s drag function [64] is employed for the relative velocity as [62]:

(9) $${\vec{V}}_{pf}=\frac{\rho_{p}d_{p}^{2}\left(\rho_{p}-\rho_{m}\right)}{18\mu_{f}f_{drag}\rho_{p}}(g-({\vec{V}}_{m}.\vec{\nabla }){\vec{V}}_{m})$$

(10) $$f_{\mathrm{drag}}=\{\begin{array}{l} 1+0.15{Re}_{}^{0.687}\left(Re\leq 1000\right)\\ 0.0183Re\left(Re>1000\right) \end{array}$$

Therefore, the drift velocity is given as:

(11) $${\vec{V}}_{dr,p}=\vec{V_{pf}}-\overset{n}{\underset{k=1}{\sum }}\left(\frac{\phi_{k}\rho_{k}}{\rho_{m}}{\vec{V}}_{fk}\right)$$

### 3.1. Nanofluid Thermo-Physical Properties

The properties of water-Al_2_O_3_ nanofluid i.e., density [10,65], heat capacity [66], effective dynamic viscosity [67], and effective thermal conductivity [67] are given as:

(12) $$\rho_{\mathrm{nf}}=\left(1-\phi \right)\rho_{f}+\phi \rho_{s}$$

(13) $${\left(\rho c_{p}\right)}_{\mathrm{nf}}=\left(1-\phi \right){\left(\rho c_{p}\right)}_{f}+\phi {\left(\rho c_{p}\right)}_{s}$$

(14) $$\mu_{\mathrm{nf}}=\frac{\mu_{f}}{{\left(1-\phi \right)}^{2.5}}$$

(15) $$k_{\mathrm{nf}}=1+2.72\phi +4.97\phi^{2}$$

A water-Al_2_O_3_ nanofluid with three values of volume concentrations is used in the present study, as listed in Table 1. The properties of the nanofluid are calculated based on the concentration of nanoparticles according to Equations (12)–(15).

### 3.2. Boundary Conditions and Data Reduction

Constant temperature and velocity are used at the tube inlet, while zero relative gauge pressure is used at the outlet for the fluid. The channel wall is exposed to a uniform heat flux of 5000 W/m^2^. The twisted tape rotates at three different angular velocities of $V_{\mathrm{inlet}}/r$ (named RTT1), $2V_{\mathrm{inlet}}/r$ (named RTT2), and $3V_{\mathrm{inlet}}/r$ (named RTT3) with an adiabatic surrounded wall.

The hydrothermal parameters investigated, along with the performance evaluation criterion (PEC), are defined as follows [23,62]:

(16) $$D_{h}=\frac{4A}{P}$$

(17) $$f_{\mathrm{ave}}=\frac{2\Delta P}{\rho U^{2}}\frac{D_{h}}{L}$$

(18) $$h_{x}=\frac{q^{\prime \prime }}{T_{w}-T_{b}}$$

(19) $${\mathrm{Nu}}_{x}=\frac{h_{x}D_{h}}{k}$$

(20) $${\mathrm{Nu}}_{\mathrm{ave}}=\frac{1}{L}\int_{0}^{L}{\mathrm{Nu}}_{x}dx$$

(21) $$\mathrm{PEC}=\frac{\frac{\mathrm{Nu}}{{\mathrm{Nu}}_{p}}}{{\left(\frac{f}{f_{p}}\right)}^{\frac{1}{3}}}$$

## 4. Numerical Procedure

ANSYS FLUENT software (version 18.0) is employed to resolve the conventional governed equations using a coupled algorithm to solve the velocity–pressure coupling, and a second-order upwind scheme is used to discretize the convection terms. The convergence criteria are set to 10-6 for both, whereas it is 10-11 for the energy equation.

### 4.1. Grid Study

**Figure 2:**
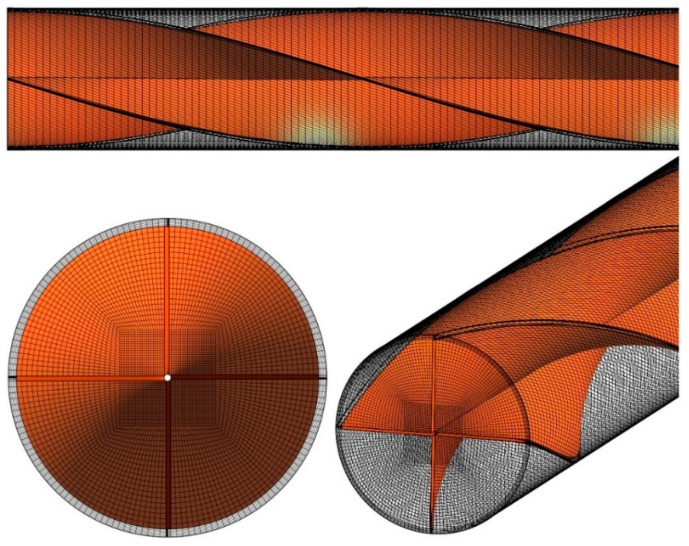
The computational mesh for Case 5.

An overview of the computational grid is illustrated in Figure 2 for Case 5 in Figure 1. Considering the advantages of structured mesh over unstructured mesh, such as higher quality of results, higher convergence speed, and convergence ease, and also the use of fewer cells, a structured mesh was generated in this study. ANSYS Meshing software was used to create the current mesh. 

**Figure 3:**
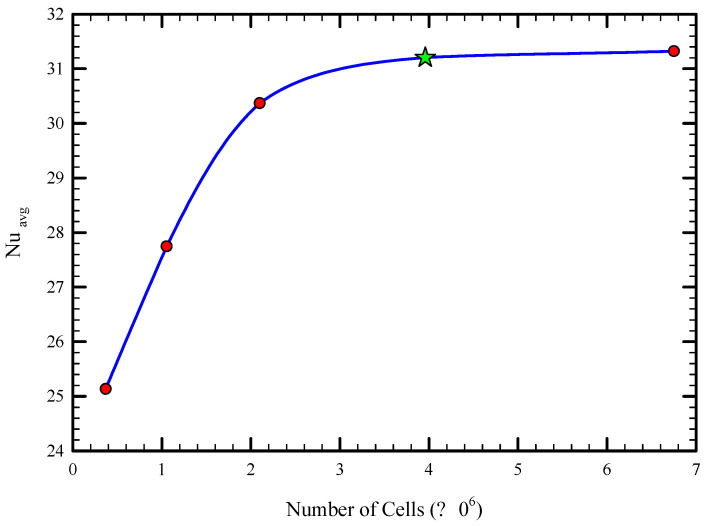
The grid independency analysis for the average Nusselt number for Case 5.

To ensure the non-dependence behavior of the results from the computational grid, the average Nusselt number for various numbers of elements was examined. Figure 3 displays the variation of the average Nusselt number for a different number of cells for the Reynolds number of 1000 for Case 5 for the volume concentration of 3%. As shown, after reducing the cell size four times, the change in the average Nusselt number is negligible (almost 0.4%), and therefore an optimal mesh size of 395,000 was selected for further analysis.

### 4.2. Code Validation with Experimental Data

**Figure 4:**
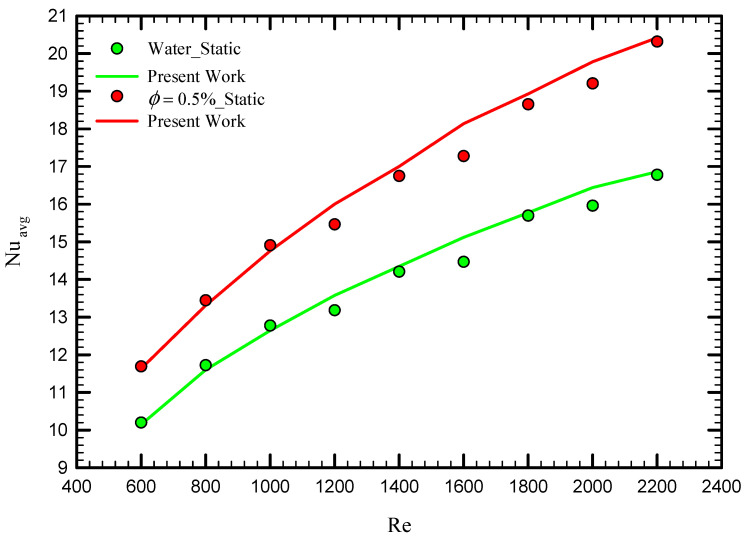
The validation study for the case of water and nanofluid compared with Qi et al. [52].

To obtain a reliable result, the experimental results of Qi et al. [52] for the average Nusselt number for laminar fluid flow in a heat exchanger using stationary twisted tape were used for comparison. They experimentally examined pure water and nanofluid fluid flow for different Reynolds numbers for a twisted tape length of 1600 mm and pitch size of 100 mm, with a width and thickness of 16 and 2 mm, respectively. Figure 4 displays the average Nusselt number for the cases of pure water for the cases of stationary twisted tapes for different Reynolds numbers. As shown, the results are in excellent agreement with the experimental data of Qi et al. [52], where the maximum difference is less than 2%.

## 5. Result and Discussion 

The numerical simulations are conducted to evaluate the thermo-hydraulic performance of laminar swirl flow through the circular tube equipped with multiple semi-twisted tapes. The importance of the number of twisted tapes, Re numbers, and nanofluid concentrations are evaluated in this research.

### 5.1. Effect of Number of Semi-Twisted Tapes

**Figure 5:**
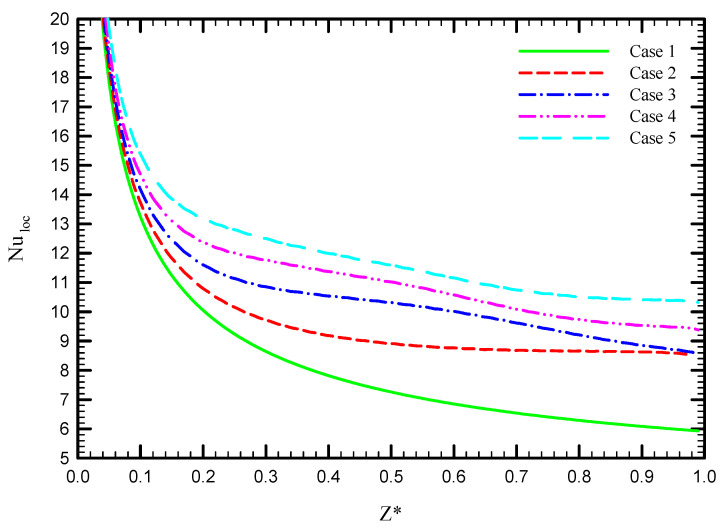
Local Nusselt number along the dimensionless tube length for the captured cases at Re = 250 φ = 0%.

Figure 5 shows the variation of local Nu number along the tube length for both the plain tube and the different multiple semi-twisted tape cases shown in Figure 1. As seen in this figure, the addition of a semi-twisted tape (i.e., Case 1) increases the local Nu number along the length of the tube. This is attributed to the twisted tape inducing a secondary flow, which provides better flow mixing patterns and a higher heat transfer rate. Increasing the number of semi-twisted tapes from N = 1 to N = 4 implies more disturbance in the flow pattern as a result of a larger perturbing surface and higher numbers of swirling flow, leading to an improvement in the local Nu number, especially within the first half of the tube length.

**Figure 6:**
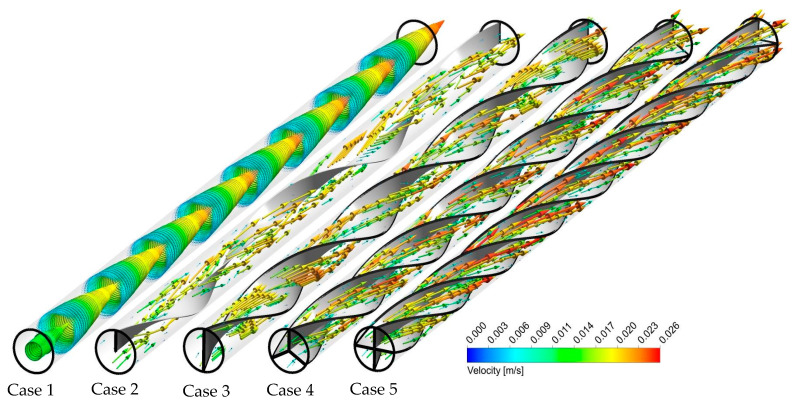
Velocity vectors for the captured cases at Re = 250 and φ = 0%.

The reason for the behavior of Nu is connected to the flow behavior under different circumstances. Figure 6 illustrates the velocity contours for the captured configurations at an Re number of 250. It is evident from this figure that only axial flow is detected in the plain tube, whereas both swirl and axial flows are recognized in the multi-channel twisted tape cases. A case with a higher number of semi-twisted tape experiences a decrease in the cross-sectional area, which improves the flow velocity. By proceeding along the tube length, more swirl flow streams are induced when the number of semi-twisted tapes is varied from N = 1 to N = 4. Multiple semi-twisted tapes generate multi-swirling flows in core and near-wall zones, causing a better fluid mixing between the core and near tube wall regions. As a result, velocity increase in the near-wall region and decreases in the core region. 

**Figure 7:**
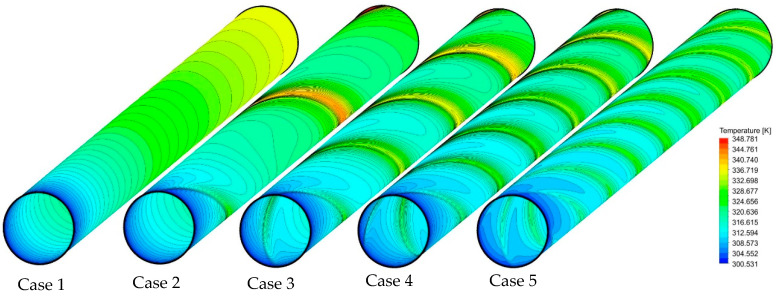
Temperature contours on the heated wall for the captured cases at Re = 250 and φ = 0%.

Figure 7 shows how increasing the number of semi-twisted tapes influences the cooling performance of the tube wall at an Re of 250. As seen in the plain tube case, there is a gradient increase in the temperature along the axial direction of the tube due to the growth in the thermal boundary layer thickness. For other cases, the presence of semi-twisted tapes has disturbed the boundary layer due to the secondary flows created in the tube. With single semi-twisted tape, although the temperature distribution on the heated wall has become more uniform, there are still some regions with local hot spots, mainly located in the middle and end of the tube. Moving from single to four semi-twisted tapes seems to be a promising trajectory towards preventing the local hot spots and providing a more uniform temperature profile along the tube.

**Figure 8:**
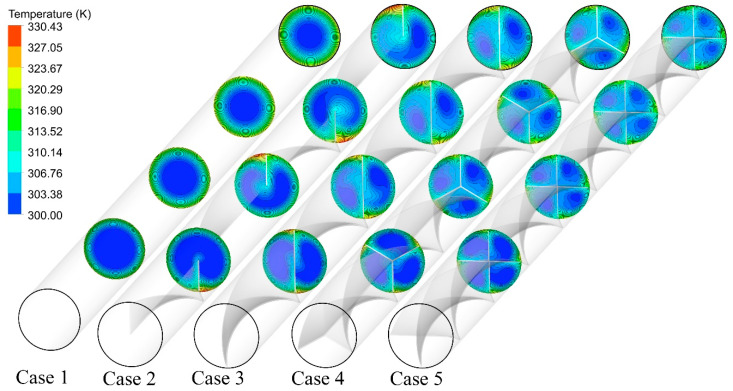
Cross-sectional temperature contours for the captured cases at Re = 250 and φ = 0%.

Figure 8 illustrates the temperature contour plots at different cross-sections for the considered configurations at an Re of 250. For the case of twisted tape with N = 1, the thermal boundary layer thickness is slightly decreased due to the better flow mixing, as opposed to the plain tube with a thick thermal boundary layer on the tube wall. However, there are some regions with rather higher thermal boundary layer thickness. The high intensity of swirl flow in cases with a higher number of semi-twisted tape (see Figure 6) results in the temperature field becoming more disordered and the temperature difference between the core and near-wall region becoming smaller as compared to a plain tube. As such, the boundary layer becomes thinner as the semi-twisted tape number increases from N = 2 to N = 4.

**Figure 9:**
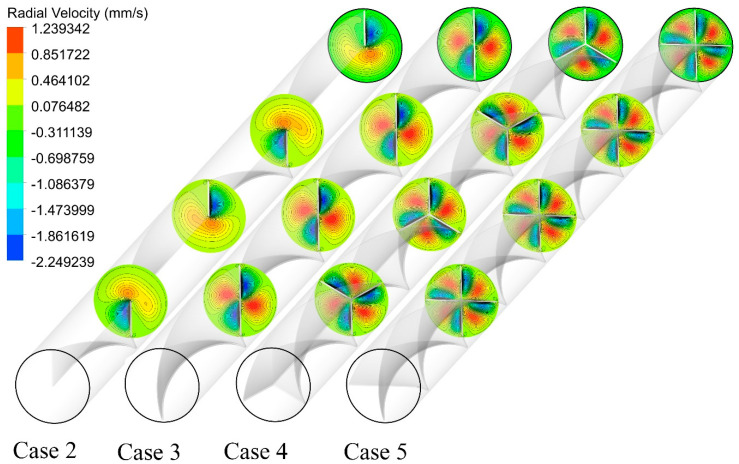
Cross-sectional radial velocity contours for the captured cases at Re = 250 and φ = 0%.

The analysis of radial and tangential velocity provides details about the role of multiple semi-twisted tape addition on the intensity of secondary flow. As seen in Figure 9, negative radial velocity is found in the vicinity of the twisted tape, while positive radial velocity is observed in the regions far from the twisted tape. Two low and two high radial velocity regions are detected in the case of N = 1, while four low and four high radial velocity regions are found in the case of N = 4. This indicates that there is a continuous movement in the fluid from the wall to the core and conversely from the core to the wall. It is also inferred that the value of radial velocity is higher in the case of higher numbers of semi-twisted tape (N = 4) as compared to that of a single semi-twisted tape case. This is the result of higher rotational movement, more centrifugal action, more efficient interruption in the thermal boundary layer, and a higher amount of swirl motion in the flow. 

**Figure 10:**
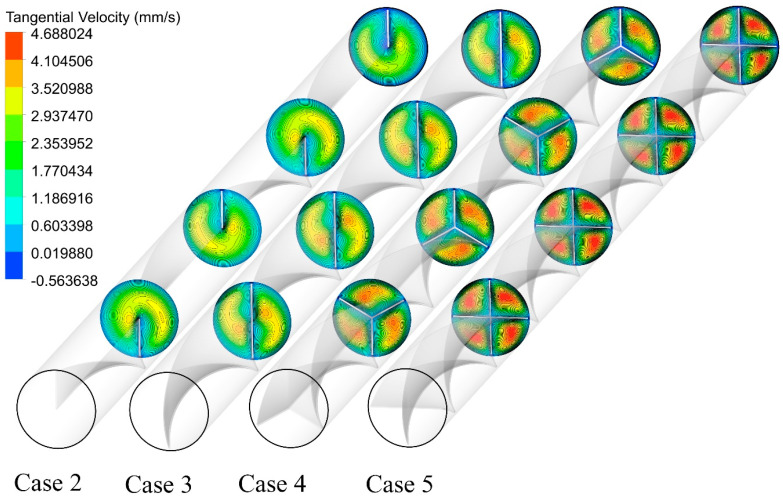
Cross-sectional tangential velocity contours for the captured cases at Re = 250 and φ = 0%.

Figure 10 shows the cross-sectional tangential velocity contours for different cases at an Re of 250. The multiple semi-twisted tapes with N = 4 generate a higher tangential velocity and enhances the heat and mass transfer between the central fluid and near-wall regions. This improves the synergy degree between the velocity fields and temperature gradient.

### 5.2. Effect of the Re Number

**Figure 11:**
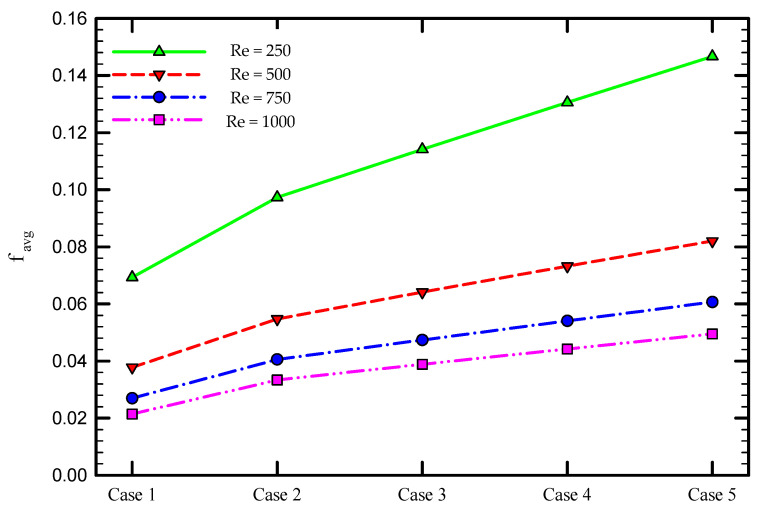
Friction coefficient for the captured cases at different Re numbers for φ = 0%.

**Figure 12:**
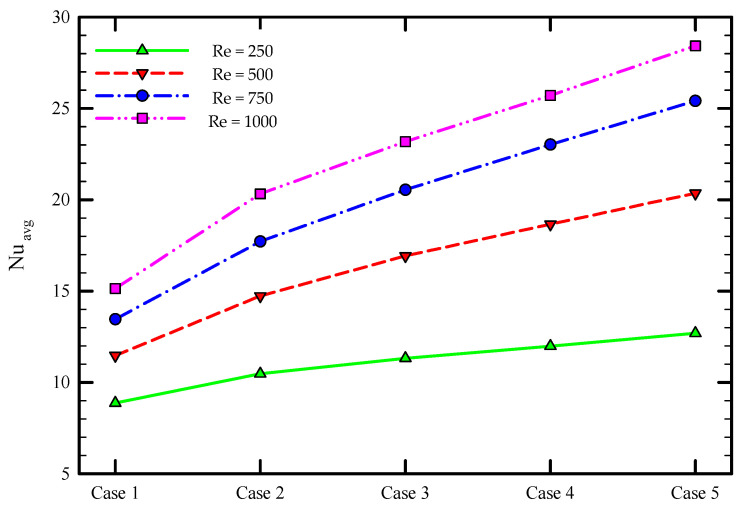
Average Nusselt number for the captured cases at different Re numbers for φ = 0%.

To assess the thermal performance of multiple semi-twisted tapes at different Re numbers, the friction coefficient and average Nu number as a function of Re number is depicted in Figure 11 and Figure 12 for the captured cases. It is found that the friction coefficient drops by increasing the Re number, as shown in Figure 11. Another observation from this figure is that the higher average friction factor is achieved when a higher number of semi-twisted tape is employed in the tube. The installation of multiple semi-twisted tapes blocks the passage of fluid flow, imposes a longer fluid flow path, and causes further friction at the tape surface. This is associated with more pumping power consumed in these cases relative to the plain tube case. The presence of flow mixing and secondary flow in addition to the primary flow is another source of larger fluid flow resistance created by multiple twisted tapes. This frictional loss enlarges by increasing the number of twisted tapes. It is evident from Figure 12 that an increase in Re number is accompanied by a rise in the Nu number for all the captured cases. This is due to the fact that moving towards a higher Re number is followed by an increase in the fluid flow velocity and swirl intensity transmitted to the flow adjacent to the tube wall, which is beneficial for effective heat dissipation. Furthermore, heat transfer enhancement in multiple twisted tapes is superior to that of single twisted tape. This is attributed to the multiple swirl flows generated by the multiple twisted tapes, leading to stronger multiple swirl intensities as well as better fluid mixing between the core fluid and the fluid near the tube wall compared to the single twisted tape case. This intense swirl flow helps the fluid washing the tube wall continuously as well as taking heat away more effectively and, as a result, increasing the average Nu number.

**Figure 13:**
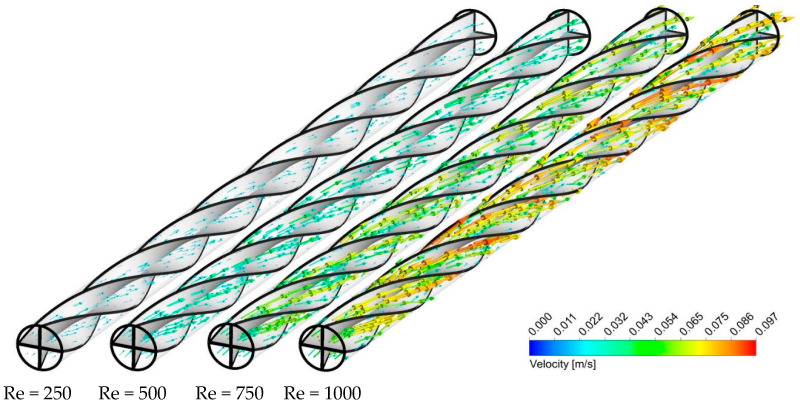
Velocity vector for Case 5 at different Re numbers and φ = 0%.

**Figure 14:**
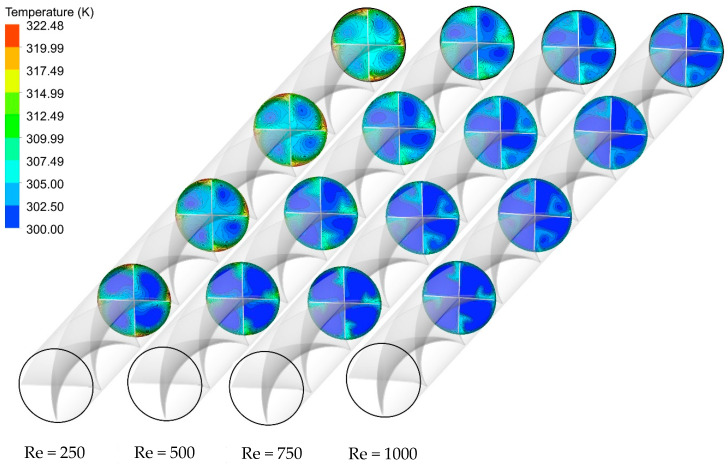
Cross-sectional temperature contours for Case 5 at different Re numbers and φ = 0%.

**Figure 15:**
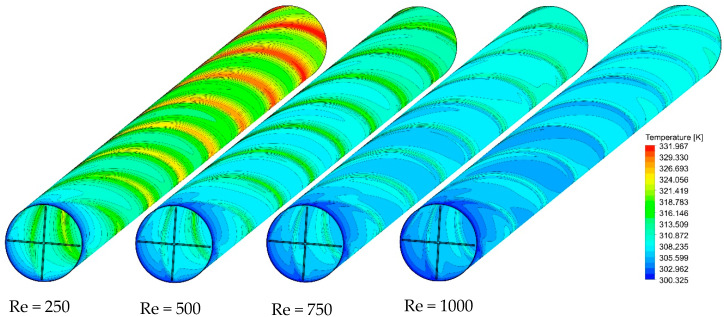
Temperature contours on the heated wall for Case 5 at different Re numbers and φ = 0%.

As a case in point, Figure 13, Figure 14 and Figure 15 show the effect of Re number on the velocity contours, cross-sectional temperature contours, and temperature contours on the heated wall for Case 5, respectively. As seen in Figure 13, as the Reynolds number increases, the fluid velocity increases throughout the length of the tube, which intensifies the swirling disturbance created by the twisted tape and the higher heat transfer rate. Moreover, the stronger flow mixing between the central and near-wall regions at higher velocity enables more effective heat dissipation from the wall and more uniform temperature distribution on the heated wall as compared to the lower Re number (Figure 14). To better understand this, the cross-sectional temperature for Case 5 is depicted for different Re numbers. As seen in this figure, the larger boundary layer thickness is induced at an Re number of 250, and switching to higher Re numbers tends to make the thermal boundary layer thickness thinner. This, together with the presence of multiple semi-twisted tapes with N = 4, is more appealing in terms of decreasing the temperature difference between the core and the near-wall region.

### 5.3. Effect of Nanofluid Concentration

**Figure 16:**
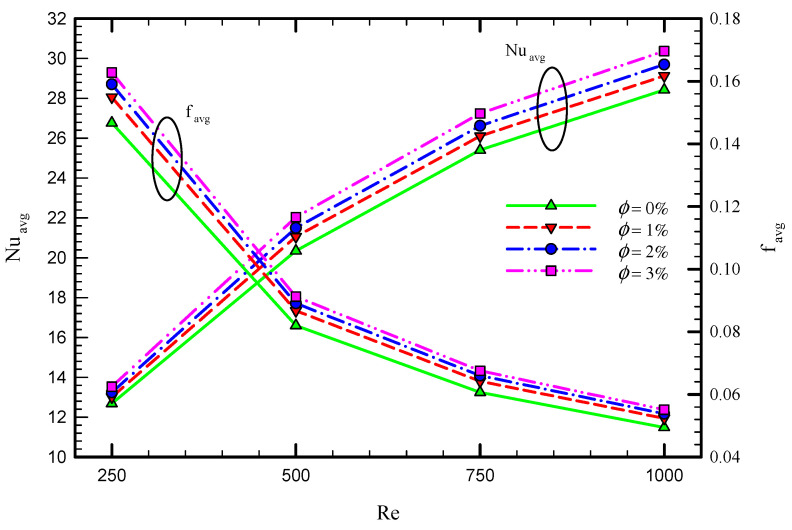
Variation of average Nu number and friction coefficient for Case 5 at different Re numbers and nanofluid volume concentrations.

The effect of the number of semi-twisted tapes and Re number is investigated on the thermo-hydraulic performance of laminar swirl flow through the circular tube. This section is devoted to the importance of nanofluid addition in the considered configurations. Figure 16 shows the variation of average Nu and friction coefficient for Case 5 at different Re numbers and nanofluid concentrations. It is found that the average Nu number and friction coefficient increases by employing nanofluid in the base fluid. The reason for the higher Nu number can be justified by the higher thermal conductivity of the nanofluid compared with the base fluid. The increase in nanofluid concentration from 0 to 3% improves the Nu number by 11.31%, 12.09%, 12.45%, and 13.24% for the Re number of 250, 500, 750, and 1000, respectively. However, this comes at the expense of a higher resistance coefficient, which is a result of high viscosity caused by increasing the nanofluid volume fraction. 

**Figure 17:**
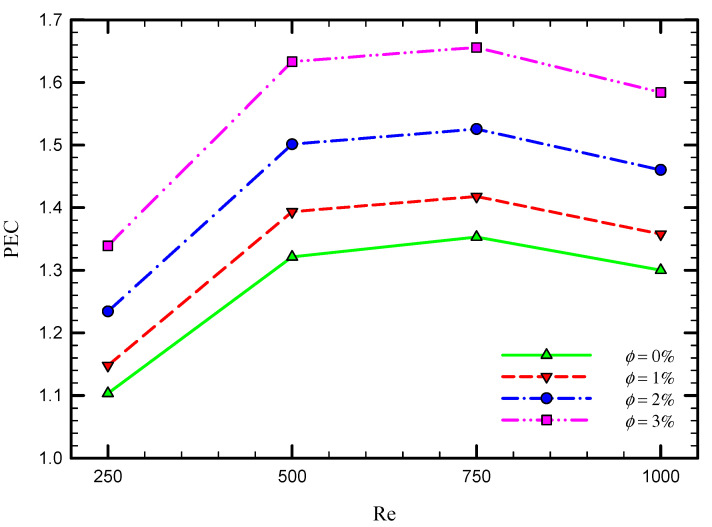
Variation of PEC with Re for Case 5 at different nanofluid volume concentrations.

As shown in Figure 16, the obtained results reveal that, although adding nanoparticles to the base fluid is beneficial in terms of enhancing the heat transfer rate, this is associated with a higher frictional resistance coefficient and pressure drop. Therefore, the performance evaluation criterion (PEC) is introduced as a quantifiable metric to assess the trade-off between the heat transfer and pressure drop. Figure 17 illustrates the variation of PEC number in terms of Re number and nanofluid volume fraction for the captured cases. As seen in this figure, PEC increases by increasing the Re number until the Re number of 750 and then reduces. Furthermore, PEC enhances using a higher volume concentration of nanoparticles. Based on the definition of PEC, the variation of PEC depends on the variation of both heat transfer and also pressure drop by adding inserts in the pipe. Depending on the operating conditions of the studied problem and also the dimensions of the system, the PEC can be reduced or enhanced at different Reynolds number, as also shown in the literature [57]. In the proposed system, the amount of increase in heat transfer compared with the increase in the pressure drop using multiple twisted tape inserts reduces in the high Reynolds number of 1000. The Maximum PEC occurs at the Reynolds number 750.

**Figure 18:**
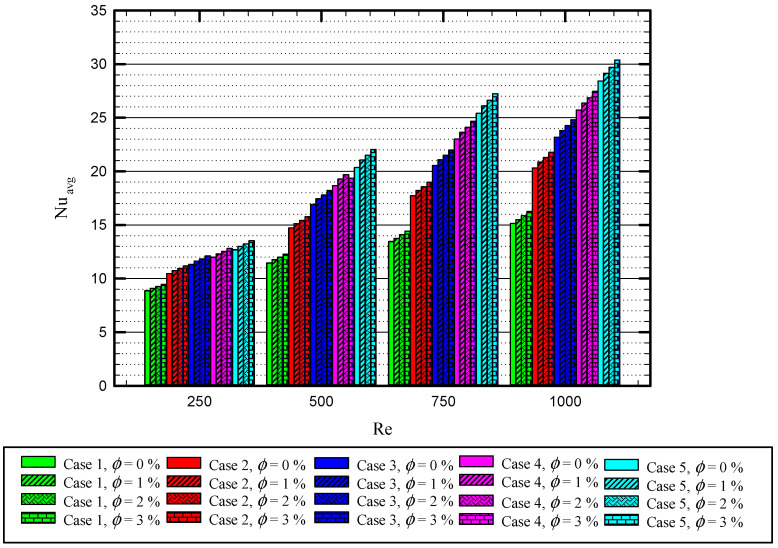
Variation of the average Nu in terms of Re for different nanoparticles concentration for Cases 2, 3, 4, and 5.

Figure 18 illustrates the relation between average Nu with Re at different nanoparticles concentration and for all the cases. Generally, the figure shows that Nu is proportional to the Re, φ, and the number of the twisted tape. As mentioned, Nu enhances using a higher Re number and nanoparticle concentration. The highest values of the average Nu is 30.2 and belongs to Case 5 for Re = 1000, φ=0.3%, while the minimum value of average Nu is 8.8 for Case 1, where Re = 250 for the pure water.

**Figure 19:**
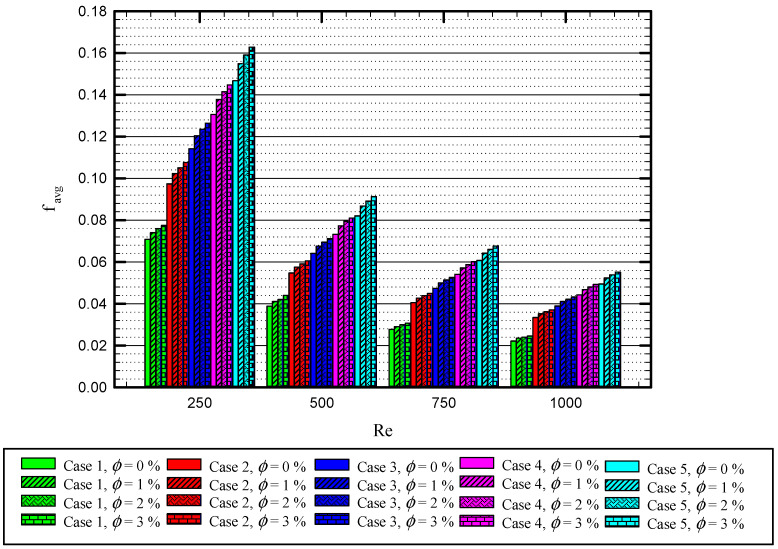
The relation between the average friction and Re at different nanoparticles concentration for Cases 1, 2, 3, 4, and 5.

Figure 19 shows the relation between the average friction value for different values of Re at different concentrations of nanoparticles for different cases. The friction factor is inversely proportional to Re; however, it increases proportionally with the number of twisted tapes. The effect of the nanoparticles is negligible for reducing the friction value. The maximum friction factor is 0.155 and belongs to Case 5 for Re = 250, φ=3%, while the minimum value of the friction factor is 0.02 for Case 1 where Re = 1000 and φ=0%.

It should be noted that because the variation of PEC is not linear, as shown in Figure 5 for Case 5, all the values of Nusselt number and friction factor are presented in this study in Figure 18 and Figure 19 to provide better understanding of the PEC behavior for all the proposed systems. 

**Figure 20:**
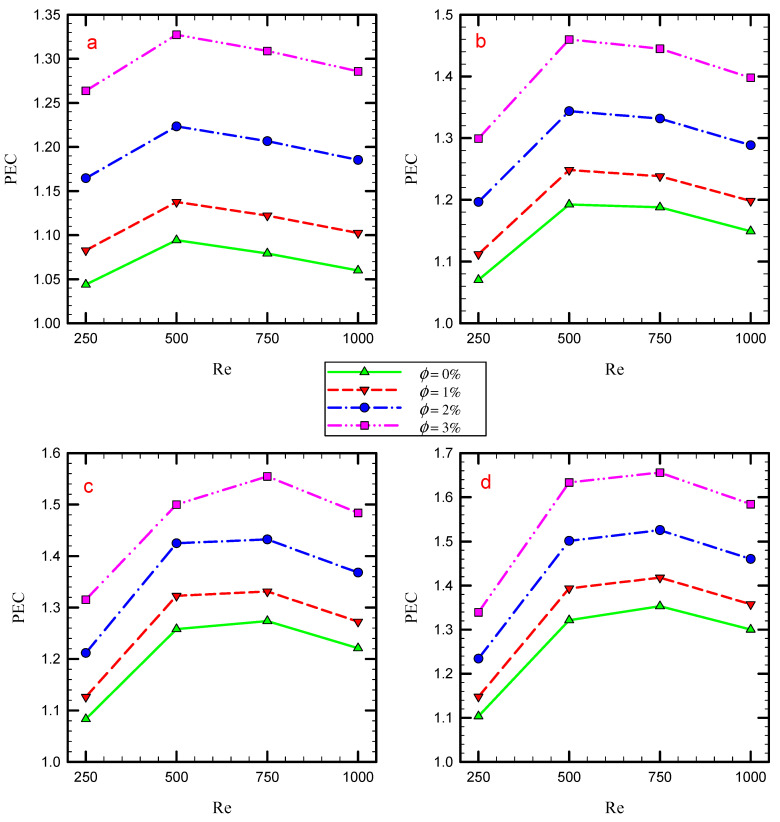
The relation between the PEC and Re at different nanoparticles concentration for Cases (**a**) 2, (**b**) 3, (**c**) 4, and (**d**) 5.

Figure 20 illustrates the effect of Re, φ, and the number of twisted tapes on the PEC. The maximum value of PEC occurs in nanoparticles concentration of 2% for the Re of 1000. The PEC shows a proportional relationship with the Re because Nu increases and friction factor decreases for a higher Re and results in a higher PEC factor. Both Nu and friction factor increase for a higher number of twisted tape inserts. As shown, the PEC also shows a proportional relationship with the number of semi-twisted tape inserts. This means that the effect of increasing the number of twisted tape inserts on the Nusselt number is higher than that for the friction factor.

## 6. Conclusions

Heat transfer improvement and friction factor characteristics of laminar nanofluid (Al_2_O_3_/water) flow in a 3D circular tube integrated with multiple semi-twisted tapes were investigated numerically. The range of nanoparticle volume fractions of 0, 1, 2, and 3%; the range of Reynolds numbers of 250, 500, 750 and 1000; and the number of the semi-twisted tapes of 0, 1, 2, 3, 4 were examined comprehensively. The computational results conclude that increasing the twisted tape number enhance both heat transfer and pressure drop; however, its effect on the heat transfer enhancement is more pronounced. The following key outcomes were achieved:

- The higher average friction factor is reached when a higher number of semi-twisted tapes are applied in the tube. The average Nusselt number, x, increases from 15 to 28.5 and the average friction factor enhances from 0.155 to 0.052 by increasing the number of the semi-twisted tapes from 0 to 4 for the Reynolds number of 1000 for the base fluid.
- Increasing the Reynolds number enhances heat transfer performance while it reduces the friction factor. By using 4 semi-twisted tapes, the average Nusselt number increases from 12.5 to 28.5, while the friction factor reduces from 0.155 to 0.052 when the Reynolds number increases from 250 to 1000 for the base fluid.
- Increasing the nanoparticle concentration results in a higher Nusselt number and friction factor. For this case at the Reynolds number of 1000, the increase in nanofluid concentration from 0 to 3% improves the average Nusselt number and friction factor by 13.24% and 3%, respectively.
- The highest PEC is 1.66 and belongs to the Reynolds number of 750 for the system using four semi-twisted tape inserts where the volume fraction of nanoparticles is 3%.

## Figures and Tables

**Table 1:** Water-Al_2_O_3_ nanofluid thermo-physical properties.

Nanoparticle Properties (d_p_ = 25 nm)				
	ρ (kg/m^3^)	$c_{p}$ (J/kgK)	k (W/mK)	μ (kg/ms)
	3880	733	36	-
Nanofluid properties				
ϕ=0%	998.200	4182.00	0.600000	0.001000
ϕ=1%	1027.018	4147.51	0.616618	0.001089
ϕ=2%	1055.836	4113.02	0.633833	0.001199
ϕ=3%	1084.654	4078.53	0.651644	0.001334

## Data Availability

Data sharing not applicable.

## Nomenclature

A	area [m^2^]	Greek symbols	
c_p_	specific heat transfer [J/kg.K]	ϑ	kinematic viscosity [m^2^/s]
f	friction coefficient	μ	dynamic viscosity [kg/m.s]
f_0_	friction coefficient of plain channel	ρ	density [kg/m^3^]
F	body force [N]	ϕ	nanofluid concentration
g	gravitational acceleration [m/s^2^]	θ	dimensionless temperature
h	heat transfer coefficient [W/m^2^K]	Subscripts	
k	thermal conductivity [W/m.K]	ave	average
n	number of phases	b	bulk
p	pressure [Pa]	m	mixture
q″	heat flux [w/m^2^]	f	fluid
Re	Reynolds number	i	inner tube
T	temperature [K]	o	outer tube
V	velocity vector [m/s]	nf	nanofuid
V_dr_	drift velocity	Abbreviations	
V_f_	fluid velocity	Co-STT	co-swirling twisted tapes
V_p_	particle velocity	Counter-STT	counter-swirling twisted tapes
		PHE	plain heat exchanger
		IT	inner tube
		OT	outer tube

## References

[1] Cai W., Liu F., Xie J., Liu P., Tuo J. (2017). A tool for assessing the energy demand and efficiency of machining systems: Energy benchmarking. Energy.

[2] Liu X., Rao R., Shi J., He J., Zhao Y., Liu J., Du H. (2021). Effect of oxygen vacancy and A-site-deficiency on the dielectric performance of BNT-BT-BST relaxors. J. Alloys Compd..

[3] Bujang A., Bern C., Brumm T. (2016). Summary of energy demand and renewable energy policies in Malaysia. Renew. Sustain. Energy Rev..

[4] Ju Y., Shen T., Wang D. (2020). Bonding behavior between reactive powder concrete and normal strength concrete. Constr. Build. Mater..

[5] Bellos E., Tzivanidis C. (2019). Alternative designs of parabolic trough solar collectors. Prog. Energy Combust. Sci..

[6] Kumar R., Chand P. (2018). Performance prediction of extended surface absorber solar air collector with twisted tape inserts. Sol. Energy.

[7] Du X., Qiu J., Deng S., Du Z., Cheng X., Wang H. (2021). Flame-retardant and solid-solid phase change composites based on dopamine-decorated BP nanosheets/Polyurethane for efficient solar-to-thermal energy storage. Renew. Energy.

[8] Gong X., Wang F., Wang H., Tan J., Lai Q., Han H. (2017). Heat transfer enhancement analysis of tube receiver for parabolic trough solar collector with pin fin arrays inserting. Sol. Energy.

[9] Zhang K., Huo Q., Zhou Y.-Y., Wang H.-H., Li G.-P., Wang Y.-W., Wang Y.-Y. (2019). Textiles/metal–Organic frameworks composites as flexible air filters for efficient particulate matter removal. ACS Appl. Mater. Interfaces.

[10] Duan Z., Yin Q., Li C., Dong L., Bai X., Zhang Y., Yang M., Jia D., Li R., Liu Z. (2020). Milling force and surface morphology of 45 steel under different Al_2_O_3_ nanofluid concentrations. Int. J. Adv. Manuf. Technol..

[11] Amina B., Miloud A., Samir L., Abdelylah B., Solano J. (2016). Heat transfer enhancement in a parabolic trough solar receiver using longitudinal fins and nanofluids. J. Therm. Sci..

[12] Gao T., Li C., Jia D., Zhang Y., Yang M., Wang X., Cao H., Li R., Ali H.M., Xu X. (2020). Surface morphology assessment of CFRP transverse grinding using CNT nanofluid minimum quantity lubrication. J. Clean. Prod..

[13] Liu Y., Wei Z., Zhong B., Wang H., Xia L., Zhang T., Duan X., Jia D., Zhou Y., Huang X. (2021). O-, N-Coordinated single Mn atoms accelerating polysulfides transformation in lithium-sulfur batteries. Energy Storage Mater..

[14] Wang P., Li Z., Xie Q., Duan W., Zhang X., Han H. (2021). A passive anti-icing strategy based on a superhydrophobic mesh with extremely low ice adhesion strength. J. Bionic Eng..

[15] Kumaresan G., Sudhakar P., Santosh R., Velraj R. (2017). Experimental and numerical studies of thermal performance enhancement in the receiver part of solar parabolic trough collectors. Renew. Sustain. Energy Rev..

[16] Hamidi S.T. (2020). A Novel Application for Parabolic Trough Solar Collector Based on Helical Receiver Tube and Nano-Fluid with a Solar Tracking Mechanism. Eng. Technol. J..

[17] Chen X., Wang D., Wang T., Yang Z., Zou X., Wang P., Luo W., Li Q., Liao L., Hu W. (2019). Enhanced photoresponsivity of a GaAs nanowire metal-semiconductor-metal photodetector by adjusting the fermi level. ACS Appl. Mater. Interfaces.

[18] Wang X., Li C., Zhang Y., Ding W., Yang M., Gao T., Cao H., Xu X., Wang D., Said Z. (2020). Vegetable oil-based nanofluid minimum quantity lubrication turning: Academic review and perspectives. J. Manuf. Process..

[19] Nakhchi M., Esfahani J. (2020). CFD approach for two-phase CuO nanofluid flow through heat exchangers enhanced by double perforated louvered strip insert. Powder Technol..

[20] Jaramillo O., Borunda M., Velazquez-Lucho K., Robles M. (2016). Parabolic trough solar collector for low enthalpy processes: An analysis of the efficiency enhancement by using twisted tape inserts. Renew. Energy.

[21] Borunda M., Garduno-Ramirez R., Jaramillo O. (2019). Optimal operation of a parabolic solar collector with twisted-tape insert by multi-objective genetic algorithms. Renew. Energy.

[22] Wang M., Yang L., Hu B., Liu J., He L., Jia Q., Song Y., Zhang Z. (2018). Bimetallic NiFe oxide structures derived from hollow NiFe Prussian blue nanobox for label-free electrochemical biosensing adenosine triphosphate. Biosens. Bioelectron..

[23] Erfanian Nakhchi M., Rahmati M. (2020). Turbulent Flows inside Pipes Equipped with Novel Perforated V-Shaped Rectangular Winglet Turbulators: Numerical Simulations. J. Energy Resour. Technol..

[24] Jaisankar S., Radhakrishnan T., Sheeba K. (2009). Experimental studies on heat transfer and friction factor characteristics of forced circulation solar water heater system fitted with helical twisted tapes. Sol. Energy.

[25] Sui M., Li C., Wu W., Yang M., Ali H.M., Zhang Y., Jia D., Hou Y., Li R., Cao H. (2021). Temperature of grinding carbide with castor oil-based MoS_2_ nanofluid minimum quantity lubrication. J. Therm. Sci. Eng. Appl..

[26] Gao T., Zhang X., Li C., Zhang Y., Yang M., Jia D., Ji H., Zhao Y., Li R., Yao P. (2020). Surface morphology evaluation of multi-angle 2D ultrasonic vibration integrated with nanofluid minimum quantity lubrication grinding. J. Manuf. Process..

[27] Nakhchi M., Rahmati M. (2021). Entropy generation of turbulent Cu–water nanofluid flows inside thermal systems equipped with transverse-cut twisted turbulators. J. Therm. Anal. Calorim..

[28] Bellos E., Tzivanidis C. (2018). Enhancing the performance of evacuated and non-evacuated parabolic trough collectors using twisted tape inserts, perforated plate inserts and internally finned absorber. Energies.

[29] Jafar K.S., Sivaraman B. (2017). Performance characteristics of parabolic solar collector water heater system fitted with nail twisted tapes absorber. J. Eng. Sci. Technol..

[30] Wang Y., Li C., Zhang Y., Yang M., Li B., Jia D., Hou Y., Mao C. (2016). Experimental evaluation of the lubrication properties of the wheel/workpiece interface in minimum quantity lubrication (MQL) grinding using different types of vegetable oils. J. Clean. Prod..

[31] Zhang C., Wang D., Ren K., Han Y., Zhu Y., Peng X., Deng J., Zhang X. (2016). A comparative review of self-rotating and stationary twisted tape inserts in heat exchanger. Renew. Sustain. Energy Rev..

[32] Arunachala U. (2020). Experimental Study with Analytical Validation of Energy Parameters in Parabolic Trough Collector with Twisted Tape Insert. J. Sol. Energy Eng..

[33] Zhang Y., Li C., Jia D., Zhang D., Zhang X. (2015). Experimental evaluation of the lubrication performance of MoS2/CNT nanofluid for minimal quantity lubrication in Ni-based alloy grinding. Int. J. Mach. Tools Manuf..

[34] Nakhchi M.E., Esfahani J. (2019). Sensitivity analysis of a heat exchanger tube fitted with cross-cut twisted tape with alternate axis. J. Heat Transf..

[35] Lim K.Y., Hung Y.M., Tan B.T. (2017). Performance evaluation of twisted-tape insert induced swirl flow in a laminar thermally developing heat exchanger. Appl. Therm. Eng..

[36] Mwesigye A., Bello-Ochende T., Meyer J.P. (2016). Heat transfer and entropy generation in a parabolic trough receiver with wall-detached twisted tape inserts. Int. J. Therm. Sci..

[37] Sajadi B., Soleimani M., Akhavan-Behabadi M.A., Hadadi E. (2020). The effect of twisted tape inserts on heat transfer and pressure drop of R1234yf condensation flow: An experimental study. Int. J. Heat Mass Transf..

[38] Kurnia J.C., Chaedir B.A., Sasmito A.P. (2020). Laminar convective heat transfer in helical tube with twisted tape insert. Int. J. Heat Mass Transf..

[39] Khoshvaght-Aliabadi M., Feizabadi A. (2020). Performance intensification of tubular heat exchangers using compound twisted-tape and twisted-tube. Chem. Eng. Process. Process Intensif..

[40] Bahiraei M., Mazaheri N., Aliee F. (2019). Second law analysis of a hybrid nanofluid in tubes equipped with double twisted tape inserts. Powder Technol..

[41] Dalkılıç A.S., Türk O.A., Mercan H., Nakkaew S., Wongwises S. (2019). An experimental investigation on heat transfer characteristics of graphite-SiO_2_/water hybrid nanofluid flow in horizontal tube with various quad-channel twisted tape inserts. Int. Commun. Heat Mass Transf..

[42] He W., Toghraie D., Lotfipour A., Pourfattah F., Karimipour A., Afrand M. (2020). Effect of twisted-tape inserts and nanofluid on flow field and heat transfer characteristics in a tube. Int. Commun. Heat Mass Transf..

[43] Bhuiya M.M.K., Roshid M.M., Talukder M.M.M., Rasul M.G., Das P. (2020). Influence of perforated triple twisted tape on thermal performance characteristics of a tube heat exchanger. Appl. Therm. Eng..

[44] Eisapour M., Eisapour A.H., Hosseini M.J., Talebizadehsardari P. (2020). Exergy and energy analysis of wavy tubes photovoltaic-thermal systems using microencapsulated PCM nano-slurry coolant fluid. Appl. Energy.

[45] Pandya N.S., Shah H., Molana M., Tiwari A.K. (2020). Heat transfer enhancement with nanofluids in plate heat exchangers: A comprehensive review. Eur. J. Mech. B Fluids.

[46] Shamsul Azha N.I., Hussin H., Nasif M.S., Hussain T. (2020). Thermal Performance Enhancement in Flat Plate Solar Collector Solar Water Heater: A Review. Processes.

[47] Shi M., Wang B., Shen Y., Jiang J., Zhu W., Su Y., Narayanasamy M., Angaiah S., Yan C., Peng Q. (2020). 3D assembly of MXene-stabilized spinel ZnMn2O4 for highly durable aqueous zinc-ion batteries. Chem. Eng. J..

[48] Chen X., Jiang B., Wang D., Li G., Wang H., Wang H., Wang F., Wang P., Liao L., Wei Z. (2021). Gate-tunable the interface properties of GaAs–WSe2 (1D–2D) vdWs heterojunction for high-responsivity, self-powered photodetector. Appl. Phys. Lett..

[49] Zhang J., Wu W., Li C., Yang M., Zhang Y., Jia D., Hou Y., Li R., Cao H., Ali H.M. (2020). Convective Heat Transfer Coefficient Model under Nanofluid Minimum Quantity Lubrication Coupled with Cryogenic Air Grinding Ti–6Al–4V. Int. J. Precis. Eng. Manuf. Green Technol..

[50] Gao T., Li C., Zhang Y., Yang M., Jia D., Jin T., Hou Y., Li R. (2019). Dispersing mechanism and tribological performance of vegetable oil-based CNT nanofluids with different surfactants. Tribol. Int..

[51] Yan X., Huang X., Chen Y., Liu Y., Xia L., Zhang T., Lin H., Jia D., Zhong B., Wen G. (2021). A theoretical strategy of pure carbon materials for lightweight and excellent absorption performance. Carbon.

[52] Qi C., Wang G., Yan Y., Mei S., Luo T. (2018). Effect of rotating twisted tape on thermo-hydraulic performances of nanofluids in heat-exchanger systems. Energy Convers. Manag..

[53] Sundar L.S., Singh M.K., Punnaiah V., Sousa A.C. (2018). Experimental investigation of Al_2_O_3_/water nanofluids on the effectiveness of solar flat-plate collectors with and without twisted tape inserts. Renew. Energy.

[54] Sheikholeslami M., Jafaryar M., Li Z. (2018). Second law analysis for nanofluid turbulent flow inside a circular duct in presence of twisted tape turbulators. J. Mol. Liq..

[55] He X., Zhang T., Xue Q., Zhou Y., Wang H., Bolan N.S., Jiang R., Tsang D.C. (2021). Enhanced adsorption of Cu (II) and Zn (II) from aqueous solution by polyethyleneimine modified straw hydrochar. Sci. Total Environ..

[56] Farshad S.A., Sheikholeslami M. (2019). Simulation of exergy loss of nanomaterial through a solar heat exchanger with insertion of multi-channel twisted tape. J. Therm. Anal. Calorim..

[57] Saysroy A., Eiamsa-Ard S. (2017). Enhancing convective heat transfer in laminar and turbulent flow regions using multi-channel twisted tape inserts. Int. J. Therm. Sci..

[58] Farshad S.A., Sheikholeslami M. (2019). FVM modeling of nanofluid forced convection through a solar unit involving MCTT. Int. J. Mech. Sci..

[59] Bianco V., Manca O., Nardini S. (2014). Performance analysis of turbulent convection heat transfer of Al_2_O_3_ water-nanofluid in circular tubes at constant wall temperature. Energy.

[60] Riahi A., Khamlich S., Balghouthi M., Khamliche T., Doyle T.B., Dimassi W., Guizani A., Maaza M. (2020). Study of thermal conductivity of synthesized Al_2_O_3_-water nanofluid by pulsed laser ablation in liquid. J. Mol. Liq..

[61] Alsarraf J., Moradikazerouni A., Shahsavar A., Afrand M., Salehipour H., Tran M.D. (2019). Hydrothermal analysis of turbulent boehmite alumina nanofluid flow with different nanoparticle shapes in a minichannel heat exchanger using two-phase mixture model. Phys. A Stat. Mech. Appl..

[62] Albojamal A., Vafai K. (2017). Analysis of single phase, discrete and mixture models, in predicting nanofluid transport. Int. J. Heat Mass Transf..

[63] Manninen M., Taivassalo V., Kallio S. (1996). On the Mixture Model for Multiphase Flow.

[64] Schiller L. (1933). A drag coefficient correlation. Zeit. Ver. Deutsch. Ing..

[65] Khanafer K., Vafai K. (2011). A critical synthesis of thermophysical characteristics of nanofluids. Int. J. Heat Mass Transf..

[66] Bianco V., Manca O., Nardini S., Vafai K. (2015). Heat Transfer Enhancement with Nanofluids.

[67] Maïga S.E.B., Palm S.J., Nguyen C.T., Roy G., Galanis N. (2005). Heat transfer enhancement by using nanofluids in forced convection flows. Int. J. Heat Fluid Flow.

